# Correction: Ye et al. Evaluation of the Brewing Characteristics, Digestion Profiles, and Neuroprotective Effects of Two Typical Se-Enriched Green Teas. *Foods* 2022, *11*, 2159

**DOI:** 10.3390/foods12061339

**Published:** 2023-03-22

**Authors:** Yuanyuan Ye, Jiangling He, Zhijun He, Na Zhang, Xiaoqing Liu, Jiaojiao Zhou, Shuiyuan Cheng, Jie Cai

**Affiliations:** 1 National R&D Center for Se-Rich Agricultural Products Processing, Hubei Engineering Research Center for Deep Processing of Green Se-Rich Agricultural Products, School of Modern Industry for Selenium Science and Engineering, Wuhan Polytechnic University, Wuhan 430023, China; 2 Key Laboratory for Deep Processing of Major Grain and Oil, Ministry of Education, Hubei Key Laboratory for Processing and Transformation of Agricultural Products, Wuhan Polytechnic University, Wuhan 430023, China; 3 Shenzhen Key Laboratory of Marine Biotechnology and Ecology, College of Life Sciences and Oceanography, Shenzhen University, Shenzhen 518055, China; 4 Hubei Key Laboratory of Nutritional Quality and Safety of Agro Products, Wuhan 430064, China

The authors wish to make the following corrections to their published paper [1]. The authors sincerely apologize for any inconvenience caused and state that the scientific conclusions of the paper are unaffected. This correction was approved by the Academic Editor. The original publication has also been updated.

**Text Correction**

There was a typing error included on page 5. In Section 3.1, the first paragraph, line four, the numerical value “7.53” should be changed to “7.5”, the numerical value “5.95 ± 0.24 µg/g” should be revised to “4.33 ± 0.18 µg/g”, and the numerical value “0.79 ± 0.02 µg/g” should be revised to “0.58 ± 0.01 µg/g”.

**Errors in Table**

In the original publication, there was a typing error in Table 1 (page 6). The numerical value “0.79 ± 0.02” should be replaced with “0.58 ± 0.01”, and “5.95 ± 0.24” should be replaced by “4.33 ± 0.18”. The corrected Table 1 is presented below.

## Figures and Tables

**Table 1:** Comparison of the main components of ESYL and ZYMJ (μg/g).

Chemical Constituents	ESYL (μg/g)	ZYMJ (μg/g)
Se	0.58 ± 0.01	4.33 ± 0.18
Tea polyphenols	16.74 ± 0.69	21.50 ± 0.47
Caffeine	4.43 ± 0.09	5.41 ± 0.31
Free amino acids	7.02 ± 0.03	6.76 ± 0.06
Soluble sugar	19.18 ± 1.07	11.65 ± 1.15
Water extracts	42.59 ± 0.16	46.23 ± 0.21

Values are means ± SD from three independent triplicate experiments.
