# Supplementary material for: Evaluation of the Brewing Characteristics, Digestion Profiles, and Neuroprotective Effects of Two Typical Se-Enriched Green Teas
Source: Foods. 2022 Jul 21;11(14):2159. doi: 10.3390/foods11142159 (PMC9318317; doi:10.3390/foods11142159)
Supplement: Supplementary file 1 [file foods-11-02159-s001.zip › foods-1823327-supplementary.pdf]

foods

Supplementary Materials

Evaluation of the Brewing Characteristics, Digestion Profiles, and Neuroprotective Effects of Two Typical Se-Enriched Green Teas

Yuanyuan Ye [1,2], Jiangling He [1,*], Zhijun He [1,3], Na Zhang [1], Xiaoqing Liu [1,2], Jiaojiao Zhou [1], Shuiyuan Cheng [1] and Jie Cai [2,4,*]

[1] National R&D Center for Se-rich Agricultural Products Processing, Hubei Engineering Research Center for Deep Processing of Green Se-rich Agricultural Products, School of Modern Industry for Selenium Science and Engineering, Wuhan Polytechnic University, Wuhan 430023, China; yyyuan0127@163.com (Y.Y.); hezj@email.szu.edu.cn (Z.H.); 12617@whpu.edu.cn (N.Z.); 18871027325@163.com (X.L.); jiaojiaozhou@whpu.edu.cn (J.Z.); s_y_cheng@sina.com (S.C.)

[2] Key Laboratory for Deep Processing of Major Grain and Oil, Ministry of Education, Hubei Key Laboratory for Processing and Transformation of Agricultural Products, Wuhan Polytechnic University, Wuhan 430023, China

[3] Shenzhen Key Laboratory of Marine Biotechnology and Ecology, College of Life Sciences and Oceanography, Shenzhen University, Shenzhen 518055, China

[4] Hubei Key Laboratory of Nutritional Quality and Safety of Agro Products, Wuhan 430064, China

* Correspondence: hejiangling@whpu.edu.cn (J.H.); caijievip@whpu.edu.cn (J.C.)

2.3. Determination of the content of tea polyphenols, caffeine, free amino acids, soluble sugar and water extracts

2.3.1. Determination of phenolic content

Total polyphenols contents in tea infusions were measured in accordance with national standard GB/T 8313-2018. Folin-Ciocalteu reagent (5 mL, 10% *v*/*v*) was then placed into test tubes containing standards, water (blank), or tea infusion samples (1 mL, diluted 10 times in water). After waiting 5 min, sodium carbonate (Na_2CO_3) solution (4 mL, 7.5 % w/v) was then added. Absorbance was measured at 765 nm after the mixture had stood at room temperature in the dark for 60 min. The results were calculated with reference to a standard curve of gallic acid (GA, 0-50 μg/mL).

2.3.2. Determination of caffeine

The amount of caffeine in tea infusion was determined in accordance with Chinese standard GB/T 8312-2013. Tea infusion samples (10 mL, diluted 2-folds in water) were added to 100-mL volumetric flasks. Then, hydrochloric acid (HCl, 4 mL, 0.01 M) and basic lead acetate (1 mL, 50% *w*/*v*) were added and diluted with water to a final volume of 100 mL. The solution was blended, rested for 10 min, and filtered through filter paper. 25 mL of the filtrate was mixed with 0.1 mL of sulphuric acid (H_2SO_4, 4.5 M), diluted to 50 mL with water, mixed and left for 10 min. After filtration, absorbance values of the filtrate were recorded at 274 nm.

2.3.3. Determination of free amino acids

According to Chinese standard GB/T 8314-2013, the free amino acids content in the tea infusion was examined. In 25-mL cuvettes, 1 mL of tea infusion, standards, or water (blank) was mixed with 0.5 mL of phosphate buffer (pH = 8.0) and 0.5 mL of ninhydrin (2% *w*/*v*), and samples were then heated in a boiling water bath for 15 min. Before measuring absorption at 570 nm, the obtained mixtures were volumed up to the scale with water after cooling. The concentration of free amino acids in the samples was determined from a standard curve generated with L-Theanine (0-0.6 mg/mL) solutions.

2.3.4. Determination of soluble sugar

As instructed by the plant soluble sugar content detection kit, the content of soluble sugar in tea infusion was determined. Briefly, 0.2 mL of tea infusion samples, standards, and water (blank) were mixed with 0.2 mL of water, followed by the addition of a 0.1-mL working solution and a 1-mL concentrated sulfuric acid. Following that, the reaction mixture was incubated in a 95 °C water bath. After 10 min, the obtained solutions were cooled down to room temperature and absorbances were recorded at 620 nm. The amount of soluble sugar in each sample was calculated from a standard curve of glucose (0-0.2 mg/mL) solutions.

2.3.5. Determination of water extracts

50 mL of tea infusion sample was transferred into an aluminum box with a known mass and kept in the oven at 105 °C for 3 h. The aluminum box was cooled in a desiccator and then weighed on an analytical balance. Water extracts content was expressed by mass fraction and calculated using the following equation:

$$\text{Water extracts content (\%)} = (m_1 - m_2)/m_0 \times 100\% \quad (1)$$

where m_1 was the mass of the tea infusion sample and the aluminum box after drying; m_2 was the mass of the aluminum box before drying; and m_0 was the mass of the tea sample.

2.6. Determination of free radical scavenging activity

The antioxidant capacity of tea infusions brewed under different conditions and digested samples collected at different time points was determined by using 2,2-Diphenyl-1-picrylhydrazyl (DPPH) and 2,2'-azinobis(3-ethylbenzthiazoline-6-sulfonic acid (ABTS) assays as described previously with slight modifications [1,2].

The DPPH reagent prepared in absolute ethanol (0.1 mM, 100 μL) was added to the diluted sample (100 μL) and the mixture was then let to stand for 30 min in the dark at room temperature. The scavenging activity was quantified by measuring the absorption at 517 nm. Absolute ethanol was used as the blank during the 517 nm absorbance measurement. The control group was prepared by mixing absolute ethanol (100 μL) with DPPH (100 μL). The following equation was used to express the results as the percentage of DPPH radicals that was scavenged:

$$\text{DPPH radical scavenging rate (\%)} = (1 - (Abs_{sample} - Abs_{blank})/Abs_{control})) \times 100\% \quad (2)$$

where Abs_{sample} was the absorbance of the sample, Abs_{blank} was the absorbance of the blank, and $Abs_{control}$ was the absorbance of the control.

The ABTS radical cation (ABTS·+) was made by the reaction of the ABTS solution (7 mM) and potassium persulfate (2.45 mM) in equal volumes, and the mixture was placed in the dark for 12-16 h before use. As-prepared ABTS·+ stock solution was diluted to an absorbance of 0.70 (± 0.02) at 734 nm in absolute ethanol. The ABTS·+ working solution (200 μL) obtained was mixed with 10 μL of the diluted sample or absolute ethanol, and the absorbances were read at 734 nm after 6 min of room temperature incubation. The following equation was used to express the results as the proportion of the ABTS radical that was scavenged:

$$\text{ABTS radical scavenging rate (\%)} = (1 - (Abs_{sample}/Abs_{control})) \times 100\% \quad (2)$$

where Abs_{sample} was the absorbance of the sample and $Abs_{control}$ was the absorbance of the control.

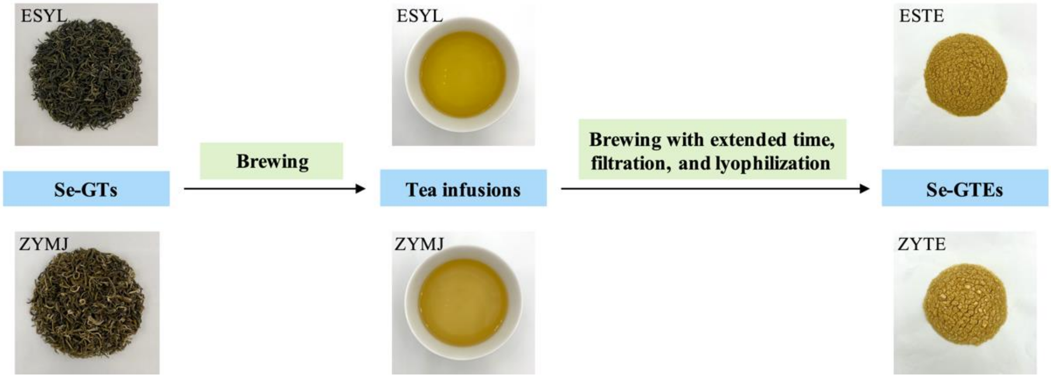

Figure S1. Flowchart for preparation process of tea infusions and Se-GTEs.

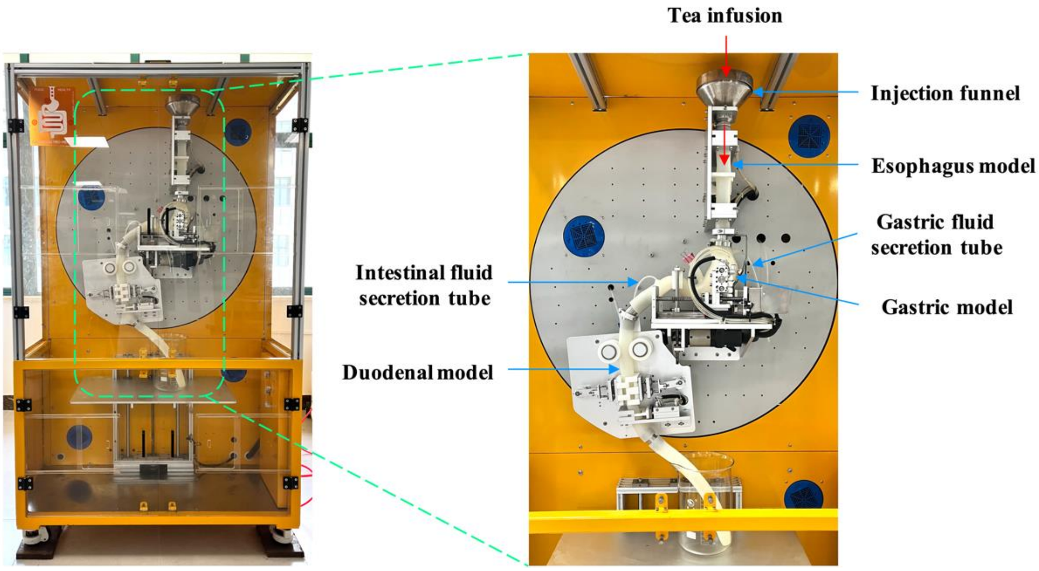

Figure S2. The structure of the dynamic stomach-intestine IV (DHSI-IV) model.

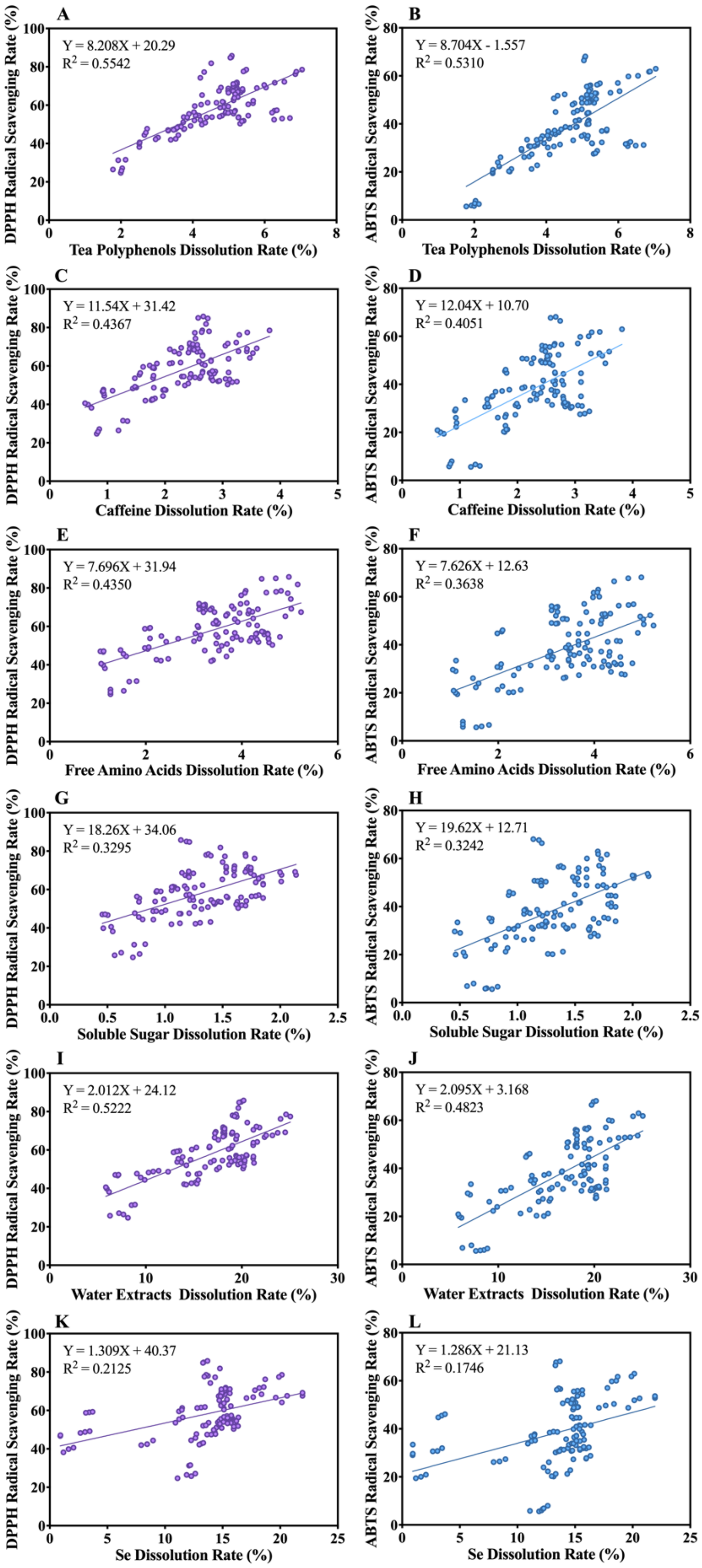

Figure S3. Correlations of antioxidant activity (DPPH and ABTS assay) with the leaching rate of tea polyphenols (**A**,**B**), caffeine (**C**,**D**), free amino acids (**E**,**F**), soluble sugar (**G**,**H**), water extracts (**I**,**J**) and Se (**K**,**L**).

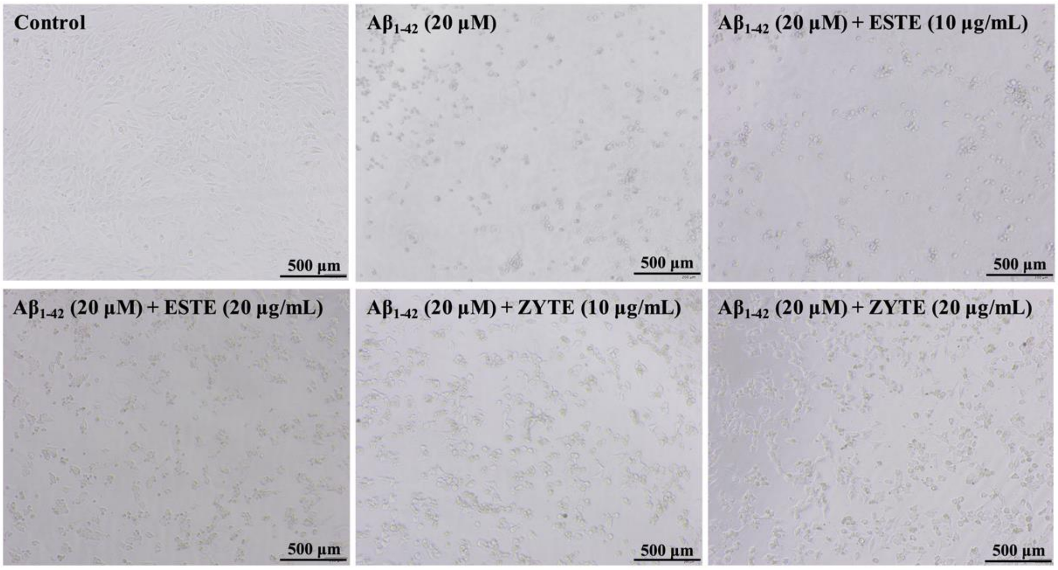

Figure S4. The morphology of HT22 cells were monitored after treating with different concentrations of Se-TEs (10, 20 μM) for 2 h and A$\beta_{1\text{-}42}$ (20 μM) for 22 h.

References

1. Sui, X.; Dong, X.; Zhou, W. Combined effect of pH and high temperature on the stability and antioxidant capacity of two anthocyanins in aqueous solution. *Food Chemistry* **2014**, *163*, 163-170, doi:10.1016/j.foodchem.2014.04.075.
2. Fernando, C.D.; Soysa, P. Extraction Kinetics of phytochemicals and antioxidant activity during black tea (*Camellia sinensis L.*) brewing. *Fernando and Soysa Nutrition Journal* **2015**, *14*, 74, doi:10.1186/s12937-015-0060-x.
